# Estimated Gestational Age From Infant's Foot Length in Japanese

**DOI:** 10.7759/cureus.32991

**Published:** 2022-12-27

**Authors:** Shingo Niimi, Takuro Kimura, Reo Saiki, Hiroshi Kanda, Yuki Okamatsu, Maki Goto, Shuichi Yatsuga

**Affiliations:** 1 Department of Pediatrics, Iizuka Hospital, Iizuka, JPN; 2 Department of Pediatrics, Tagawa Hospital, Tagawa, JPN; 3 Department of Obstetrics and Gynecology, Iizuka Hospital, Iizuka, JPN; 4 Department of Pediatrics, Fukuoka University, Fukuoka, JPN

**Keywords:** estimate calculation formula of gestational age, japanese, infants, gestational age, foot length

## Abstract

Aim

In developed countries including Japan, gestational age (GA) is predicted by the last menstrual period (LMP) and/or fetal ultrasound. In some developing countries, GA is predicted by infant's foot length (FL). Pregnant women who did not have pregnancy check-up is not infrequent in Japan, therefore there are sometimes opportunities to estimate the GA from infants after the delivery. The aim of this study is to determine the estimated GA formula from infant's FL in Japanese.

Methods

This study was a prospective cohort study. Infants between May 2021 and August 2021 at Iizuka Hospital and Tagawa Hospital or transferred from other hospitals within 24 hours of birth were collected. GA was determined using LMP and/or fetal ultrasound. The infant's FL was measured with a digital caliper within 24 hours of birth. The relationship between FL and GA was analyzed by simple regression analysis to determine the coefficient of determination (R^2^). The infant's FL of males and females, infant's FL of preterm and term, and infant's FL of low birth weight and appropriate weight infants were performed by the *t*-test as independent samples. A statistically significant difference was p < 0.05. Statistical analysis was performed using JMP Pro 16 (SAS Institute Japan Co., Ltd., Minato-ku, Tokyo).

Results

Ninety of the 135 infants were enrolled. The average GA was 38.2 ± 1.8 weeks, the average infant's FL was 7.230 ± 0.411 centimeter (cm), and the range of the infant's FL was 5.385 to 8.089 cm. The estimated GA formula, *GA* = 18.49 + 0.27 x *infant's FL *(R^2^ = 0.39), was determined.

Conclusions

We determined the estimated GA formula from the infant's FL. There are some limitations and care should be taken in the use.

## Introduction

Gestational age (GA) predictions are based on (1) the last menstrual period (LMP), (2) fetal ultrasound with measurements of crown-rump length (CRL), biparietal diameter (BPD), and femur length, and (3) the Dubowitz method and the New Ballard method. In Japan, LMP and fetal ultrasound are generally used because GA and fetal ultrasound are simple and highly accurate. But LMP assumes a menstrual cycle of 28 days, does not consider delays in ovulation, and may cause inaccuracies of one to four weeks [1]. Fetal ultrasound assumes dependent on the skill of the examiner and may cause errors. The Dubowitz method and the New Ballard method are generally not used in Japan due to significant bias of the examiners excluding infants who did not have pregnancy check-ups.

In Japan, many pregnant women have pregnancy check-ups during pregnancy, and many infants are decided at GA by LMP or fetal ultrasound. However, there are 0.23-8.06 infants who did not have pregnancy check-ups per 1000 pregnant women [2]. GA with the infants who did not have pregnancy check-ups is predicted by the Dubowitz method and the New Ballard method. One method of predicting GA is to use the infant's foot length (FL), which can be easily measured and is highly objective. Infant's FL is often used for predicting GA in some developing countries. There is a correlation between infant's FL and GA in India and Ethiopia [3-5]. There are no reports yet that correlate the infant's FL and GA in Japan. The aim of this study is to determine the estimated GA formula from the infant's FL of Japanese.

## Materials and methods

We conducted a prospective cohort study, which was performed from May 22, 2021, to August 31, 2021, at Iizuka Hospital and Tagawa Hospital, or transferred from other hospitals within 24 hours after birth to be a part of the study. The definition of appropriate birth weight infants was those with a weight between the 10th and 90th percentile of the standard Japanese weight, and the definition of low birth weight infants was those with a weight less than the 10th percentile of the standard Japanese weight [6]. Preterm infants were defined as those born between 22 weeks 0 days and less than 37 weeks 0 days gestation, and term infants were defined as those born between 37 weeks 0 days and less than 42 weeks 0 days gestation [1]. GA was determined using the LMP or fetal ultrasound with crown-rump length (CRL)/biparietal diameter (PMD)/femur length in the third trimester.

**Figure 1:**
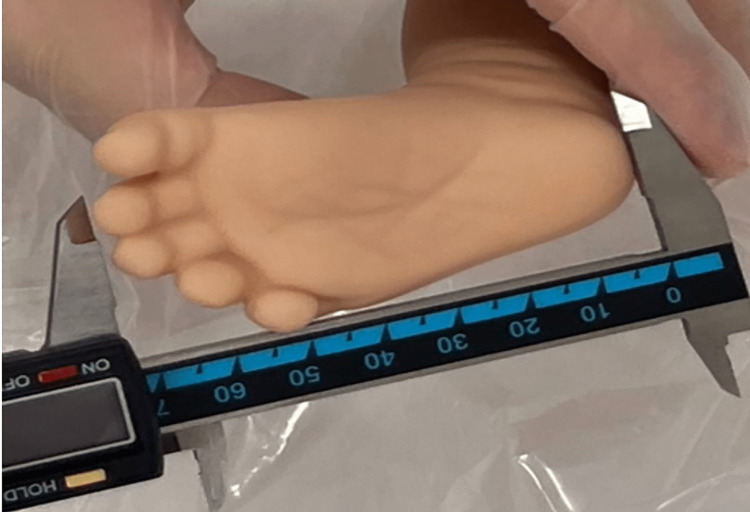
Measuring the infant's right foot length by digital caliper

The infant's FL was measured by doctors or nurses within 24 hours after birth. Five doctors (S.N, H.K, S.Y, R.S, T.K) and five nurses received explanations and were fully trained. The infant's FL was defined as the length from the center of the right heel to the longest right toe (Figure 1). Measurement of the infant's FL was unified to the right foot, measured three times with a digital caliper (Shinwa Measurement Co., Ltd., product number 19974, Niigata, Japan), and the median value was adopted. If the value was the same two out of three times, that value was used.

The relationship between the infant's FL and GA, between the infant's FL of males and GA, and between the infant's FL of females and GA was analyzed by single regression analysis to determine the coefficient of determination (R^2^). The receiver operating characteristic (ROC) curve was performed to analyze the sensitivity and specificity between the infant's FL and GA. The comparison of infant's FL of preterm and term infants and infant's FL of appropriate birth weight and low birth weight infants were analyzed by the unpaired t-test to determine the p-values. A significant difference between the groups was defined when p<0.05. Statistical analysis was performed using JMP Pro 16 (SAS Institute Japan Co., Ltd.).

This study was approved by the Ethics Committee of the Iizuka Hospital (approval number: 21065).

## Results

We collected 135 infants during the study period. Infants with no infant's FL measurements in four, infants with no pregnancy check-up in two, infants with twins in eight, and infants without appropriate birth weight or low birth weight in 31 were excluded. Finally, 90 of 135 infants were enrolled in this study, of which 35 were males (38.9%) and 55 were females (61.1%). GA ranged from 31 to 41 weeks, with 10 preterm infants (11.1%: 4 males and 6 females) and 80 term infants (88.9%: 31 males and 49 females) (Tables 1, 2). The 90 infants weighed between 1426 gm and 3454 gm, with an average weight of 2850 ± 349 gm (Table 1). There was no statistically significant difference in body weight and GA by sex (p = 0.53 and 0.55).

**Table 1:** Demographic characteristics of male and female infants Ninety infants were enrolled in this study. There were no differences between male infants and female infants in weight and gestational age (GA). Values are given as mean ± standard deviation (SD) or range in weight and GA. P-values in weight and GA for males and females are 0.52 and 0.55, respectively. N: number, min: minimum, max: maximum.

		Male	Female	Total	p
N		35	55	90	
Weight (gram)	Mean ± SD	2880.2 ± 365.1	2831.6 ± 341.6	2850.5 ± 349.7	0.52
	Min – Max	1804 – 3454	1426 – 3454	1426 – 3454	
GA (week)	Mean ± SD	38.0 ± 2.0	38.3 ± 1.7	38.2 ± 1.8	0.55
	Min – Max	32 – 41	31 – 41	31 – 41	

**Table 2:** Demographic characteristics between preterm and term infants Weight and gestational age (GA) were shown. Values are given as mean ± standard deviation (SD) or range in weight and GA. P-value in weight for preterm and term infants is < 0.0001. N: number, min: minimum, max: maximum.

		Maturity status					
		Preterm			Term		
		Male	Female	Total	Male	Female	Total
N		4	6	10	31	49	80
Weight (gram)	Mean ± SD	2103.5 ± 280.3	2270.7 ± 421.5	2203.8 ± 363.8	2980.4 ± 228.9	2900.2 ± 261.9	2931.3 ± 251.2
	Min-Max	1804 – 2376	1426 – 2548	1426 – 2548	2482 – 3454	2432 – 3454	2432 – 3454
GA (week)	Mean ± SD	33.5 ± 1.3	35 ± 2.0	34.4 ± 1.8	38.6 ± 1.1	38.7 ± 1.1	38.7 ± 1.1
	Min-Max	32–35	31 – 36	31 – 36	37 – 41	37 – 41	37 – 41

The average infant's FL of 90 infants was 7.230 ± 0.411 cm, and the range of FL was 5.385 to 8.089 cm. The average infant's FL of 35 male infants was 7.281 ± 0.418 cm (95% confidence intervals (CI): 7.138-7.307 cm), and the average infant's FL of 55 female infants was 7.197 ± 0.407 cm (95% CI: 7.087-7.307 cm). There was no statistically significant difference in infant's FL between male infants and female infants (p = 0.35) (Table 3). There was no statistically significant difference in FL between preterm and term infant's by sex (p = 0.89, 0.18) (Table 4).

**Table 3:** Foot length (FL) in male and female infants There was no difference between male infants and female infants in infant's FL. Values are given as mean ± standard deviation (SD) or range in infant's FL. The P-value of infant's FL for male and female infants was 0.35. N: number, min: minimum, max: maximum

		Male	Female	Total	p
N		35	55	90	
FL (cm)	Mean ± SD	7.281 ± 0.418	7.197 ± 0.407	7.230 ± 0.411	0.35
	Min – Max	5.97 – 8.089	5.385 – 7.886	5.385 – 8.089	

**Table 4:** Infant's foot length (FL) in preterm and term infants There was no difference between infant's FL in preterm and term infants. Values were given as mean ± standard deviation (SD) or range in infant's FL. The P-value of infant's FL for male and female infants at preterm and term was 0.89 and 0.18, respectively. The P-value of infant's FL for total preterm and total term infants was < 0.0001. N: number, min: minimum, max: maximum

		Preterm			Term		
		Male	Female	Total	Male	Female	Total
N		4	6	10	31	49	80
FL (cm)	Mean ± SD	6.706 ± 0.523	6.761 ± 0.722	6.739 ± 0.618	7.356 ± 0.347	7.250 ± 0.324	7.291 ± 0.335
	Min – Max	5.970 – 7.198	5.385 – 7.470	5.385 – 7.470	6.664 – 8.089	6.537 – 7.886	6.537 – 8.089
p		0.89			0.18		< 0.0001

We divided infants into low birth weight infants and appropriate birth weight infants. There were low birth weight infants in 12 (13.3%: 5 males and 7 females) and appropriate birth weight infants in 78 (86.7%: 30 males and 48 females). The difference in infant's FL was not statistically significant in low birth weight infants and appropriate birth weight infants between male and female infants (p = 0.81, 0.24). Infant's FL had a statistically significant difference between total low birth weight infants and total appropriate infants (p < 0.0001) (Table 5).

**Table 5:** Infant's foot length (FL) in low birth weight and appropriate birth weight infants There were no differences between male infants and female infants in low birth weight infant's FL or appropriate birth weight infant's FL. There was a significant difference between low birth weight and appropriate birth weight infants in infant's FL. Values are given as mean ± standard deviation (SD) or range in infant's FL. N: number, min: minimum, max: maximum

		Low birth weight infants			Appropriate birth weight infants		
		Male	Female	Total	Male	Female	Total
N		5	7	12	30	48	78
FL (cm)	Mean ± SD	6.799 ± 0.499	6.753 ± 0.542	6.753 ± 0.542	7.362 ± 0.351	7.267 ± 0.323	7.303 ± 0.335
	Min – Max	5.970 – 7.198	5.385 – 7.172	5.385 – 7.198	6.664 – 8.089	6.537 – 7.886	6.537 – 8.089
p		0.81			0.24		< 0.0001

**Figure 2:**
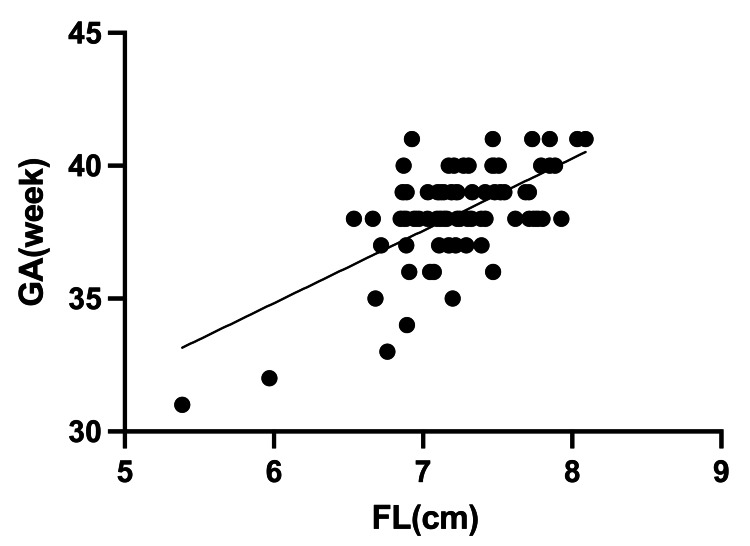
Relationship between gestational age (GA) and infant's foot length (FL) An estimated GA formula "GA = 18.49 + 0.27 x FL (R^2^ = 0.39)" was determined.

A correlation was found between GA and infant's FL, and an estimated GA formula "GA = 18.49 + 0.27 x FL (R^2^ = 0.39, Figure 2)" was determined. 

**Figure 3:**
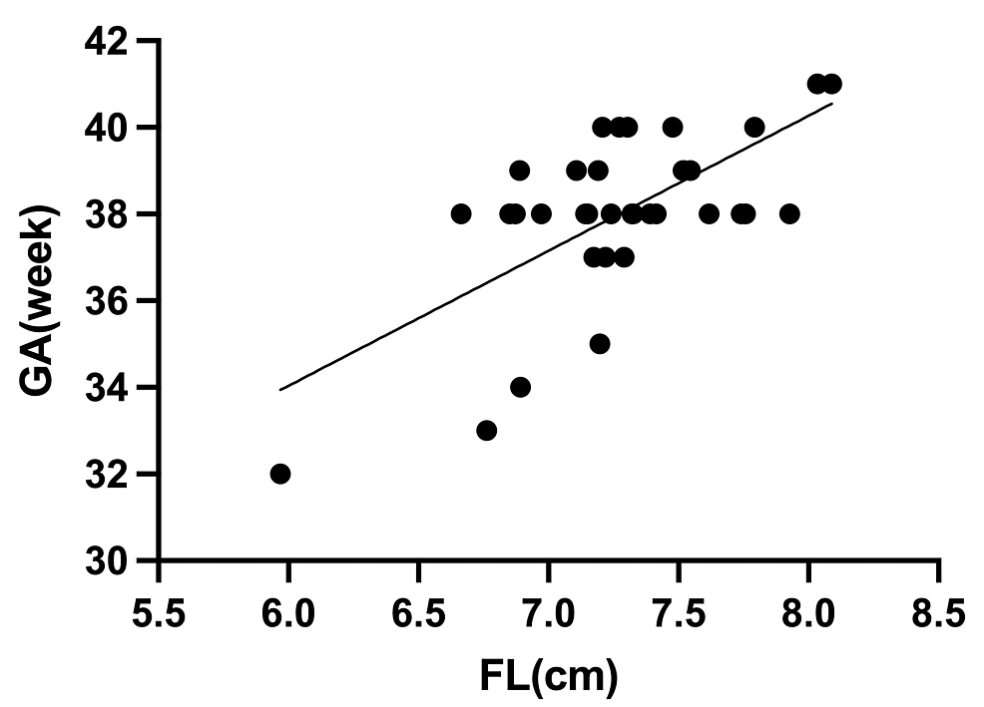
Relationship between gestational age (GA) and infant's foot length (FL) in male infants Estimated GA formula in male infants (R^2^ = 0.43; GA = 15.34 + 0.31 x FL)

**Figure 4:**
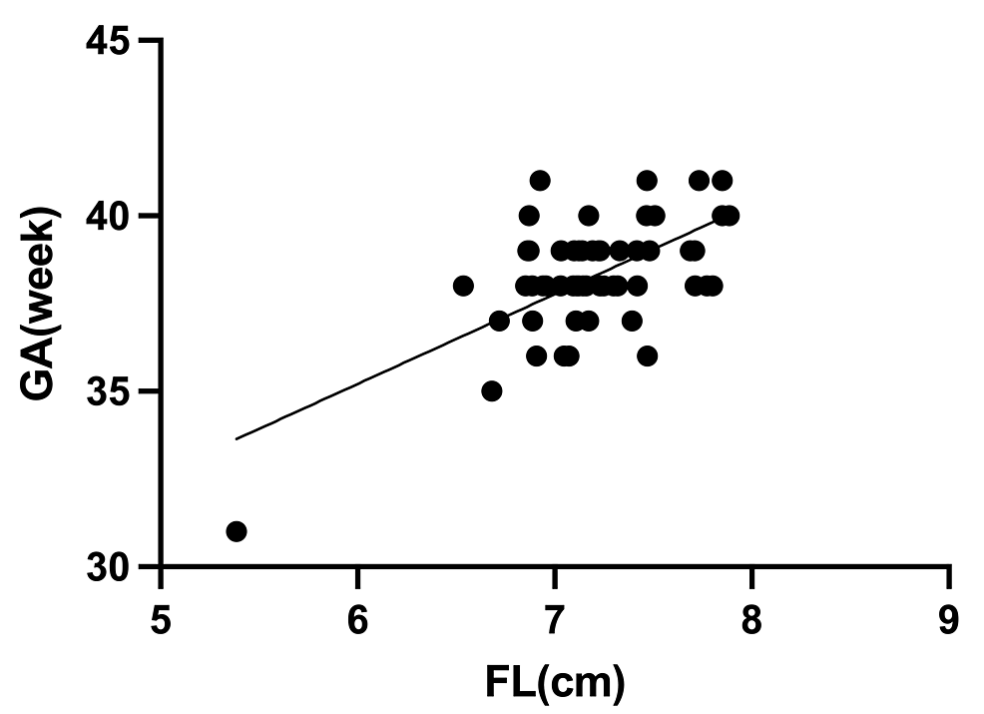
Relationship between gestational age (GA) and foot length (FL) in female infants Estimated GA formula in female infants (R^2^ = 0.39; GA = 19.86 + 0.26 x FL)

There was also a correlation between GA and FL in male infants (R^2^ = 0.43, Figure 3), yielding the correlation equation "GA = 15.34 + 0.31 x FL", and a similar correlation in female infants (R^2^ = 0.39, Figure 4), yielding the correlation equation "GA = 19.86 + 0.26 x FL".

**Figure 5:**
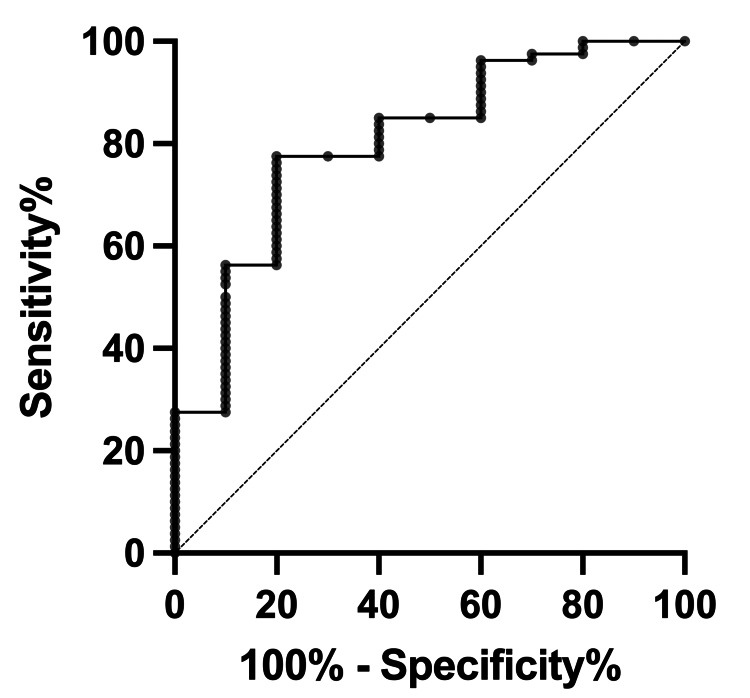
Receiver operating characteristic (ROC) curve of infant's foot length (FL) ROC showed a cutoff of 7.094 centimeters, an area under the curve (AUC) of 0.8, a sensitivity of 77.5%, and a specificity of 80%.

The receiver operating characteristic curve (ROC) for term infants showed a cutoff of 7.094 cm, an area under the curve (AUC) of 0.8, a sensitivity of 77.5%, and a specificity of 80% (Figure 5). It was clarified that there is a correlation between FL within 24 hours of birth and GA (R^2^ = 0.39).

## Discussion

The aim of this study was to determine the estimated GA formula from the infant's FL of Japanese. The results of the estimated GA formula were shown in GA = 18.49 + 2.7 x FL (R^2^ = 0.39).

This study was induced in the estimated GA formula "GA = 18.49 + 2.7 x FL (R^2^ = 0.39)". On the other hand, GA formulas were reported, such as "GA = 6.278 + 4.15 x FL (R^2^ = 0.86)" in India [3], "GA = 15.343 + 3.183 x FL (R^2^ = 0.65)" in India [4], and "GA = 3.61 + 4.5 x FL (R^2^ = 0.75)" in Ethiopia [5]. The correlation coefficient between GA and the infant's FL varied. In this study, the correlation coefficient was 0.39, which was slightly lower than in other previous reports. There was no statistically significant difference in infant's FL between male infants and female infants in this study in accordance with previous reports. There were various causes of the slightly lower correlation coefficient in this study due to differences in sample size, how to determine GA, place to measure infant's FL, and measuring instruments. In some studies, GA was determined using LMP [3], fetal ultrasound [7], or the New Ballard method [8]. Infant's FL was measured using a variety of instruments, but the most common instrument was used by the vernier caliper [4,7-10]. An infant's FL is often measured from the center of the heel to the longest toe [11]. In this study, GA was determined from LMP or ultrasound in the third trimester, and the infant's FL was measured from the center of the heel to the longest toe by a digital caliper. The measurement of infant's FL was the most common method in accordance with past reports in this study. There may be some differences in the correlation between infant's FL and GA depending on race, and the correlation may be weaker in the Japanese. The proportionality constant of Japan is smaller than that of other countries because the birth weight of Japan is lower than that of other countries, resulting in a shorter infant's FL [12]. A study from other Asian countries is required.

In developing countries, GA is sometimes predicted using the infant's FL, the Dubowitz method, and/or the New Ballard method for infants born to pregnant women who did not have a pregnancy check-up [11]. On the other hand, in Japan, GA is predicted using the Dubowitz method and/or the New Ballard method for infants born to pregnant women who did not have a pregnancy check-up, and infant's FL is not used for GA prediction. GA is usually predicted in LMP or fetal ultrasound in Japan, however, there are 0.23-8.06 infants who did not have a pregnancy check-up per 1000 pregnant women [2]. There are reports that the Dubowitz method overestimates GA and the New Ballard method underestimates GA [1]. Occasionally, infants who have not undergone pregnancy check-ups suffer severe conditions immediately after birth, then the Dubowitz method and/or the New Ballard method cannot be performed. The estimated GA formula for measuring infant' FL is conveniently useful and important, even in Japan.

The limits of this study are: (1) the number of infants was small, (2) the proportion of preterm infants was only 10%, (3) the study was limited to regional perinatal facilities and does not include cases from higher medical institutions such as university hospitals, and (4) preterm infants, perinatal infants, very low birth weight infants, etc. were not included.

## Conclusions

We determined the estimated GA formula from infant's FL. There are some limitations and care should be taken in the use. It is a simple test and can contribute to improving the treatment of infants.
